# Complete genome sequence of *Leuconostoc gelidum* subsp. *gasicomitatum* KG16-1, isolated from vacuum-packaged vegetable sausages

**DOI:** 10.1186/s40793-016-0164-8

**Published:** 2016-06-07

**Authors:** Margarita Andreevskaya, Jenni Hultman, Per Johansson, Pia Laine, Lars Paulin, Petri Auvinen, Johanna Björkroth

**Affiliations:** Institute of Biotechnology, University of Helsinki, Viikinkaari 5D, 00790 Helsinki, Finland; Department of Food Hygiene and Environmental Health, University of Helsinki, Agnes Sjöbergin katu 2, 00790 Helsinki, Finland

**Keywords:** Leuconostoc gelidum subsp. gasicomitatum, Food spoilage, Functional genome annotation, Comparative genomic analysis, Modified-atmosphere packaging, Vegetable products

## Abstract

*Leuconostoc gelidum* subsp. *gasicomitatum* is a predominant lactic acid bacterium (LAB) in spoilage microbial communities of different kinds of modified-atmosphere packaged (MAP) food products. So far, only one genome sequence of a poultry-originating type strain of this bacterium (LMG 18811^T^) has been available. In the current study, we present the completely sequenced and functionally annotated genome of strain KG16-1 isolated from a vegetable-based product. In addition, six other vegetable-associated strains were sequenced to study possible “niche” specificity suggested by recent multilocus sequence typing. The genome of strain KG16-1 consisted of one circular chromosome and three plasmids, which together contained 2,035 CDSs. The chromosome carried at least three prophage regions and one of the plasmids encoded a galactan degradation cluster, which might provide a survival advantage in plant-related environments. The genome comparison with LMG 18811^T^ and six other vegetable strains suggests no major differences between the meat- and vegetable-associated strains that would explain their “niche” specificity. Finally, the comparison with the genomes of other leuconostocs highlights the distribution of functionally interesting genes across the *L. gelidum* strains and the genus *Leuconostoc*.

**Electronic supplementary material:**

The online version of this article (doi:10.1186/s40793-016-0164-8) contains supplementary material, which is available to authorized users.

## Introduction

*Leuconostoc gelidum* is a psychrotrophic LAB commonly associated with cold-stored nutrient-rich foods of meat and vegetable origins [1–7]. According to a recently published reclassification study, this species comprises three subspecies: *L. gelidum* subsp. *gelidum**,**L. gelidum* subsp. *gasicomitatum* and *L. gelidum* subsp. *aenigmaticum* [8].

*L. gelidum* subsp. *gasicomitatum* was first isolated from spoiled MAP tomato-marinated broiler meat [2]. Later, it was found in spoiled MAP beef and pork [3, 9], and acetic-acid preserved herring [10]. Recently, it was detected in connection with the spoilage of boiled eggs preserved in brine [11], vacuum-packaged vegetable sausages [4] and minimally processed vegetable salads [5]. Depending on the product type, the spoilage is characterized by the formation of gas, slime, sour and buttery off-odors, and discoloration. Together with *L. gelidum* subsp. *gelidum**,* it belongs to the predominant microbiota at the end of shelf-life in different kinds of packaged cold-stored food products [5, 12].

Based on the previous studies of the genetic diversity and population structure of *L. gelidum* subsp. *gasicomitatum**,* some strains isolated from vegetable-based products were almost not recovered from meat-derived foods [9, 13]. This might suggest “niche-specificity” of the different strains as a consequence of their genetic differences. Alternatively, the absence of strain dissemination between vegetable- and meat-processing chains was proposed as a possible factor accounting for the phenomenon observed [9].

So far, only the complete genome of *L. gelidum* subsp. *gasicomitatum* type strain LMG 18811^T^ isolated from spoiled MAP broiler meat, has been available [14]. In this study we present the complete and annotated genome sequence for the vegetable spoilage-associated strain *L. gelidum* subsp. *gasicomitatum* KG16-1 [4]. In addition, six more vegetable strains of this organism were sequenced and partially assembled. This allowed us to compare the gene repertoires of eight *L. gelidum* subsp. *gasicomitatum* strains and all leuconostocs sequenced to date to identify: i) the genetic determinants putatively accounting for the differences in lifestyle of meat and vegetable strains of the described organism, and ii) genes that are specific for the *L. gelidum* (subsp*. gasicomitatum*) species.

## Organism information

### Classification and features

**Fig. 1:**
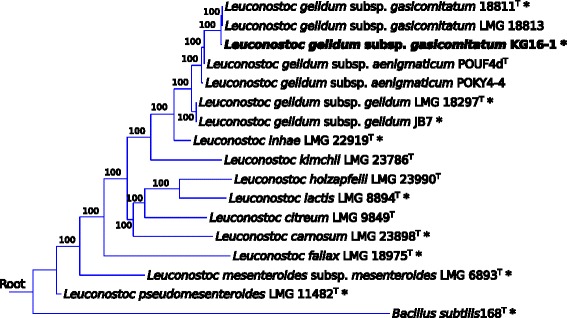
Phylogenetic tree showing the relationship of *L. gelidum* subsp. *gasicomitatum* KG16-1 to other *Leuconostoc* species. The tree was built using T-REX web server [61] based on MUSCLE [62] aligned concatenated nucleotide sequences of *atpA*, *pheS* and *rpoA* genes (Genbank identifiers are listed in Table S1 of Additional file 1). Poorly aligned positions were removed using Gblocks [63] with default parameters and the tree was inferred using RAxML program [64] with GTRCAT substitution model and *Bacillus subtilis* as an outgroup. Bootstrap analysis was performed with 500 replicates. Type strains are identified with superscripted “T” and the availability of a sequenced genome is indicated with “*”

**Fig. 2:**
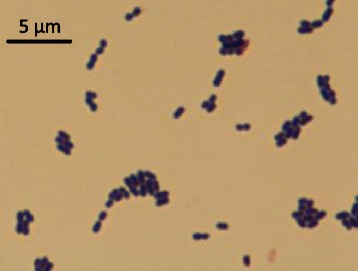
Photomicrograph of *L. gelidum* subsp. *gasicomitatum* KG16-1. The cells were grown for 48 h on the plate with MRS medium and Gram stained. The image was taken using an optical microscope with magnification 1000x

*L. gelidum* subsp. *gasicomitatum* KG16-1 is a Gram-positive, non-motile, non-sporulating, facultatively anaerobic psychrotrophic LAB, belonging to the phylum *Firmicutes* as part of the *Leuconostocaceae* family (Table 1). It was first isolated from spoiled vacuum-packaged vegetable sausages in 2006 in Helsinki, Finland (the strain was initially designated as 16-1 and then renamed into KG16-1) [4]. The sausages consisted mainly of carrot (56 %) with the addition of potato, rapeseed oil, cheese, cream, egg yolk powder and sucrose, and were stored below 8 °C for more than 20 days after cooking and packaging. The spoilage was characterized by the formation of gas and slime. Inoculation experiments showed [4] that strain KG16-1 alone is able to cause spoilage of vacuum-packaged vegetable sausages. The phylogenetic analysis based on the concatenated nucleotide sequences of *atpA*, *pheS* and *rpoA* genes (coding for ATP synthase subunit alpha, phenylalanine--tRNA ligase alpha subunit and DNA-directed RNA polymerase subunit alpha, respectively, and showed to successfully discriminate between species of the genus *Leuconostoc* [15]) clearly shows that strain KG16-1 belongs to the species *L. gelidum* subspecies *gasicomitatum* and is distinct from the subspecies *gelidum* and *aenigmaticum* (Fig. 1). According to the API 50CH carbohydrate utilization test (bioMérieux, Marcy l’Etoile, France), this bacterium is capable of fermenting a variety of carbohydrates, including hexoses, pentoses and disaccharides (Table 1). The utilization of D-galactose, N-acetylglucosamine and gentibiose is weak. Peculiarly, unlike the majority of *L. gelidum* subsp. *gasicomitatum* strains, strain KG16-1 does not ferment xylose. The cells are oval cocci, 0.5 to 1 μm in diameter (Fig. 2). Colonies formed on de Man-Rogosa-Sharpe (MRS) medium are small and greyish-white.

**Table 1:** Classification and general features of *Leuconostoc gelidum* subsp. *gasicomitatum* strain KG16-1 according to MIGS recommendations [48]

MIGS ID	Property	Term	Evidence code^a^
	Classification	Domain *Bacteria*	TAS [49]
		Phylum *Firmicutes*	TAS [50, 51]
		Class *Bacilli*	TAS [52]
		Order *Lactobacillales*	TAS [53]
		Family *Leuconostocaceae*	TAS [54]
		Genus *Leuconostoc*	TAS [55–57]
		Species *Leuconostoc gelidum*	TAS [1, 8]
		Subspecies *Leuconostoc gelidum* subsp. *gasicomitatum*	TAS [2, 8]
		Strain KG16-1	
	Gram stain	Positive	TAS [2]
	Cell shape	Coccus	TAS [2]
	Motility	Non-motile	TAS [2]
	Sporulation	Not reported	NAS
	Temperature range	Psychrotroph	TAS [2]
	Optimum temperature	25 °C	TAS [2]
	pH range; Optimum	5 – 8; 6.5	TAS [8, 58]
	Carbon source	D-glucose, D-fructose, D-mannose, L-arabinose, D-ribose, methyl D-glucoside, D-cellobiose, D-maltose, D-lactose, D-melibiose, D-raffinose, D-saccharose, D-trehalose, D-turanose	IDA
MIGS-6	Habitat	Vacuum-packaged vegetable sausages	TAS [4]
MIGS-6.3	Salinity	4 % NaCl (w/v)	TAS [8]
MIGS-22	Oxygen requirement	Facultative anaerobic	TAS [59]
MIGS-15	Biotic relationship	Free-living	NAS
MIGS-14	Pathogenicity	Non-pathogen	NAS
MIGS-4	Geographic location	Helsinki, Finland	TAS [4]
MIGS-5	Sample collection	2006	TAS [4]
MIGS-4.1	Latitude	60.19	NAS
MIGS-4.2	Longitude	24.94	NAS
MIGS-4.4	Altitude	Unknown	NAS

^a^Evidence codes - *IDA*: Inferred from Direct Assay; *TAS*: Traceable Author Statement (i.e., a direct report exists in literature); *NAS*: Non-traceable Author Statement (i.e., not directly observed for the living, isolated sample, but based on a generally accepted property for the species, or anecdotal evidence). These evidence codes are from the Gene Ontology project [60]

## Genome sequencing information

### Genome project history

Strain KG16-1 was chosen for sequencing as a representative of a vegetable-based product spoilage-associated strain of *L. gelidum* subsp. *gasicomitatum*. The manufacturer of the product was a small-size operator using vegetables as the main ingredients. No meat was handled at the plant and the other ingredients were mainly heat treated. Since this was the first wider problem reported affecting non-meat foods, and more than one product of the manufacturer was affected, a genome project was initiated. The project was carried out jointly by the Institute of Biotechnology and Department of Food Hygiene and Environmental Health, University of Helsinki, Finland. The complete genome was sequenced, fully assembled and annotated. The summary of the project information, including database identifiers, is shown in Table 2.

**Table 2:** Project information

MIGS ID	Property	Term
MIGS 31	Finishing quality	Complete
MIGS-28	Libraries used	454 fragment library (500-600 bp)
MIGS 29	Sequencing platforms	454 Sequencer with GS Flx chemistry
MIGS 31.2	Fold coverage	19 ×
MIGS 30	Assemblers	Newbler 2.0.00.20, Gap4 from Staden package
MIGS 32	Gene calling method	Prodigal, Glimmer3, tRNAscan-SE, RNAmmer, ARAGORN
	Locus Tag	LEKG
	Genbank ID	LN890331- LN890334
	GenBank Date of Release	January 20, 2016
	BIOPROJECT	PRJEB11303
MIGS 13	Source Material Identifier	KG16-1
	Project relevance	Food spoilage

### Growth conditions and genomic DNA preparation

For DNA isolation, *L. gelidum* subsp. *gasicomitatum* strain KG16-1 was grown anaerobically (Oxoid, Basingstoke, United Kingdom) in MRS broth at 25 °C overnight. DNA was extracted using a modified method [16] of Pitcher et al. [17], and the genomic DNA was mechanically sheared with a needle. The ratio of absorbance at 260 nm and 280 nm (NanoDrop spectrophotometer, Thermo Scientific, USA) as a measure of DNA purity was assessed to be ~1.8.

### Genome sequencing and assembly

Genomic DNA was sequenced using 454 Sequencer with GS Flx chemistry. The 152,753 reads obtained had an average length of 224 bp and were assembled using Newbler 2.0.00.20. In total, 149,580 reads (97.9 %) were assembled, resulting in 106 large (>500 bp) and 12 smaller (>100 bp, but < 500 bp) contigs with N50 contig size being 32,090 bp. The average sequencing coverage was 19×. The Gap4 program from the Staden package [18] was first used to design primers to the near ends (~ 150-200 bp) of the contigs and then to organize the contigs in the correct order based on the PCR products. Gaps were closed by Sanger sequencing with BigDye v3.1 chemistry and primer walking of PCR products. List of primers used can be obtained upon request. The insertion of A nucleotide (genomic position 416,796) within the mucus-binding protein-encoding gene LEKG_0412 was confirmed by linker adaptor-mediated PCR. For this, genomic DNA was digested by EcoRI restriction enzyme and then ligated to synthetic adaptors that provided binding sites for primers used during PCR amplification (5′ GCATTCACACTTAAGTTTCGTGA ′3 and 5′ TGTCGACGTTGTAAAACGACGGCCAGT 3′) and Sanger sequencing (5′ ATTAACCCTCACTAAAGGGA 3′).

### Genome annotation

Protein-coding genes were identified by Glimmer3 [19] and Prodigal [20] and their functions were predicted by RAST [21] and PANNZER [22]. The outputs of two programs for the prediction of gene coordinates as well as for gene function prediction were compared and the discrepancies were manually resolved based on the presence of potential ribosomal binding sites, similarity searches against public databases and literature data. To further improve the gene prediction, the GenePRIMP pipeline [23], which detects erroneously predicted translational start sites, broken and missing genes, was applied. To identify putative pseudogenes and possible sequencing errors, frameshift prediction was performed using GeneTack program [24] and similarity searches with proteomes of closely related species using Exonerate [25]. Bacteriocins and prophage regions were predicted with the help of BAGEL2 [26] and PHAST [27] programs, respectively. CRISPRFinder [28] was used to detect CRISPRs. rRNA, tRNA and tmRNA, were predicted with RNAmmer [29], tRNAscan-SE [30] and ARAGORN [31], respectively. Identification of transmembrane helices and signal peptides was performed with TMHMM server v. 2.0 [32] and SignalP 4.1 server [33]. Finally, the assignment of COG functional categories and prediction of Pfam domains were performed by an RPS-BLAST search (e-value threshold of 0.01 was used and the one best hit was taken into account for each gene) against the COG and Pfam databases, respectively. The genome was also checked for the presence of all core COG functions [34].

## Genome properties

**Fig. 3:**
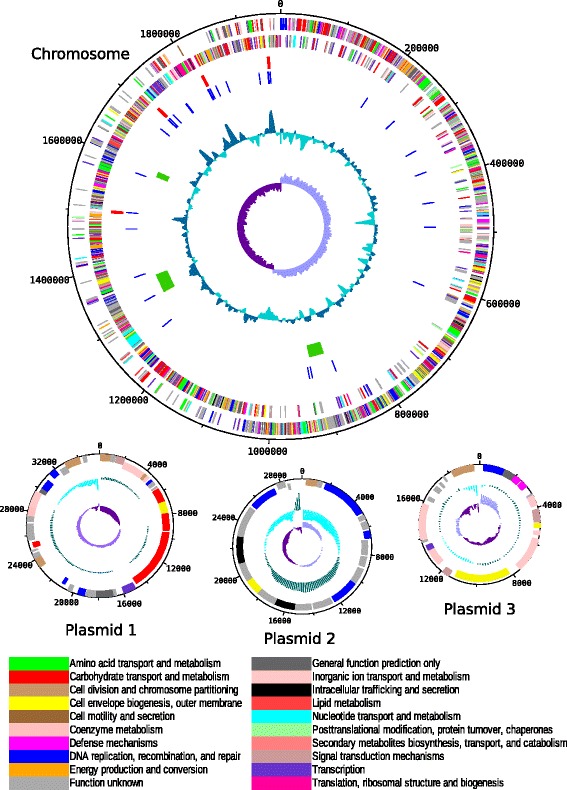
Genome map of *L. gelidum* subsp. *gasicomitatum* KG16-1. Genes are colored according to their COG functional annotations. Moving inwards, the tracks on the chromosome map represent the following features: genes on the forward strand, genes on the reverse strand, rRNAgenes (red), tRNAgenes (blue) prophages (green), GC plot (cyan), and GC skew (purple). Likewise, the tracks on the plasmid maps represent the genes on the forward and reverse strands, GC plot, and GC skew

The complete genome of *L. gelidum* subsp. *gasicomitatum* KG16-1 consisted of one circular chromosome (1,965,841 bp) and three circular plasmids (35,714 bp, 29,494 bp and 19,683 bp) (Table 3). The average GC content of the chromosome was 36.9 %. The numbers of predicted protein-coding genes were 1,944 (including 12 pseudogenes) on the chromosome and 38 (including two pseudogenes), 32 and 21 on the three plasmids, respectively (Table 4). In addition, the chromosome contained four rRNA operons (each having 5S, 16S and 23S rRNAs), 67 tRNA genes and one tmRNA gene. The putative function was assigned to 84 % of genes and COGs were assigned to 1,601 protein-coding genes (78.7 % of the total). The distribution of the protein-coding genes among COG functional categories is summarized in Table 5 and depicted in Fig. 3. All 61 core COGs of the minimal set of essential functions for a prokaryotic organism [34] were present in the genome. The genome harbored three complete prophages (LEKG_0833-0885; LEKG_1279-1342; LEKG_1560-1576) (Fig. 3). However, due to the presence of bacteriophage attachment sites inside the second prophage region, it might consist of two different prophages.

**Table 3:** Summary of genome: one chromosome and three plasmids

Label	Size (Mb)	Topology	INSDC identifier
Chromosome	1.97	Circular	LN890331
Plasmid 1	0.04	Circular	LN890332
Plasmid 2	0.03	Circular	LN890333
Plasmid 3	0.02	Circular	LN890334

**Table 4:** Genome statistics

Attribute	Value	% of Total
Genome size (bp)	2,050,732	100.0
DNA coding (bp)	1,829,560	89.2
DNA G + C (bp)	755,745	36.9
DNA scaffolds	4	
Total genes	2,115	100.0
Protein coding genes	2,035	96.2
RNA genes	80	3.8
Pseudo genes	14	0.7
Genes in internal clusters	NA	NA
Genes with function prediction	1,777	84.0
Genes assigned to COGs	1601	75.7
Genes with Pfam domains	1688	79.8
Genes with signal peptides	66	3.1
Genes with transmembrane helices	543	25.7
CRISPR repeats	0	0

**Table 5:** Number of genes associated with general COG functional categories

Code	Value	% age	Description
J	190	9.3	Translation, ribosomal structure and biogenesis
A	0	0.0	RNA processing and modification
K	144	7.1	Transcription
L	102	5.0	Replication, recombination and repair
B	0	0.0	Chromatin structure and dynamics
D	31	1.5	Cell cycle control, cell division, chromosome partitioning
V	52	2.6	Defense mechanisms
T	56	2.8	Signal transduction mechanisms
M	105	5.2	Cell wall/membrane biogenesis
N	14	0.7	Cell motility
U	21	1.0	Intracellular trafficking and secretion
O	57	2.8	Posttranslational modification, protein turnover, chaperones
C	60	2.9	Energy production and conversion
G	177	8.7	Carbohydrate transport and metabolism
E	132	6.5	Amino acid transport and metabolism
F	90	4.4	Nucleotide transport and metabolism
H	79	3.9	Coenzyme transport and metabolism
I	66	3.2	Lipid transport and metabolism
P	79	3.9	Inorganic ion transport and metabolism
Q	25	1.2	Secondary metabolites biosynthesis, transport and catabolism
R	133	6.5	General function prediction only
S	108	5.3	Function unknown
-	434	21.3	Not in COGs

The total is based on the total number of protein coding genes in the genome

## Insights from the genome sequence

### Genome functional characteristics

Based on the predicted functions in the genome, *L. gelidum* subsp. *gasicomitatum* strain KG16-1 had a very similar set of metabolic pathways to those present in strain LMG 18811^T^ and described previously [14]. Briefly, the only central carbohydrate catabolic pathway present in the genome was the phosphoketolase pathway, which classifies this bacterium as obligate heterofermentative LAB. The genome also contained genes for three alternative pyruvate utilization pathways, respiratory electron transport chain and menaquinone biosynthesis. Despite the negative reaction for xylose utilization, the required xylose catabolic genes (*xylA* and *xylB*) and putative xylose transporter (*xylP*, LEKG_1853) were present in the genome and did not contain frameshifts or premature stop codons. However, an amino acid sequence comparison of *xylA* and *xylB* between xylose-fermenting (according to API 50CH test) *L. gelidum* subsp. *gasicomitatum* strains (LMG 18811^T^, C120c, KSL4-2 and PL111) and KG16-1 showed that in KG16-1, these genes carried two (V168A, S260G) and four (S108L, L128H, D199N, G319E) amino acid substitutions, respectively, which were not present in other xylose-utilizing strains. In addition, KG16-1 was lacking the ortholog of another xylose transporter *xylT* (e.g. gene LEGAS_1062 in LMG 18811^T^), which was present in other strains. D-Ala--D-Ala ligase gene *ddl* (LEKG_0342) contained Phe^261^ in its active site, which indicates a resistance to vancomycin [35]. The same was found for all other leuconostocs sequenced to date, when sequences of *ddl* genes were aligned. Unlike the genome of strain LMG 18811^T^, strain KG16-1 contained three plasmids. The first plasmid contained the putative type I galactan catabolic gene cluster (LEKG_1953-1960), similar to the cluster present in *Leuconostoc mesenteroides* subsp. *mesenteroides*ATCC 8293 [36], and heavy metal resistance genes. Type I galactan is a structural polysaccharide comprising pectin, which is one of the major components of plant cell walls. Hence, the ability to degrade this polysaccharide would be beneficial for the growth in a plant environment. The second plasmid harbored a Type II restriction-modification (RM) system and conjugal transfer genes, while the third plasmid carried an RM system (the type is unclear) and heavy-metal resistant genes. In addition to the plasmids, the chromosome harbored at least two RM enzymes of type II (LEKG_0442 and LEKG_0445).

### Genomic comparison between *L. gelidum* subsp. *gasicomitatum* strains and other *Leuconostoc* species

The comparison of gene contents (more precisely, their protein translations) was performed for *L. gelidum* subsp. *gasicomitatum* KG16-1 and 40 other leuconostocs (assembly accession numbers are listed in Table S2 of Additional file 1). They comprised 34 sequenced and annotated genomes available at the NCBI database by September 2015, including the complete genome of *L. gelidum* subsp. *gasicomitatum*LMG 18811^T^*.* In addition, we sequenced six strains of *L. gelidum* subsp. *gasicomitatum* (C120c, C122c, KSL4-2, PB1a, PB1e, PL111) associated with spoilage of other vegetable-based food products (Table 6). The sequencing was done using Illumina HiSeq2500 platform (paired-end library with the read length of 101 + 101 bp), the draft genomes were assembled with Velvet 1.2.08 [37] and annotated by RAST [21]. The important details on the draft genomes, such as genome size, fold coverage, number of contigs and predicted CDSs, are given in Table S3 of Additional file 1.

**Table 6:** The presence/absence of selected genes in *L. gelidum* strains

Gene name	LMG 18811^T^	KG16-1	C120c	C122c	KSL4-2	PB1a	PB1e	PL111	LMG 22919^a^	LMG 18297^T^	JB7	Other *Leuconostoc*s
Lacticin biosynthesis genes	-	+	+^b^	-	-	-	-	-	-	-	-	-
Polyketide biosynthesis cluster	+	-	+	+	+	+	-	+	+^b,c^	-	-	-
Pyruvate oxidase	+	-	+	+	+	+	+	+	+	+	+	Present in 15 other leuconostocs
Biofilm formation genes	+	-	+	+	+	+	+	+	+^b,c^	+	+	*L. mesenteroides* KFRI-MG, *L. mesenteroides* subsp*. mesenteroides* J18*,* *L. pseudomesenteroides* LMG 11482^T^
Collagen-binding protein	+	-	+	+	+	+	+	+	+^b,c^	+	+	Present in all four *L. pseudomesenteroides* strains
Xylose-proton symporter	+	-	+	+	+	+	+	+	+^b,c^	+	+	Present in 15 other leuconostocs
Mucus-binding protein	+	+^b^	+	+	+	+	+	+	+^b,c^	-	-	-
Accessory Sec system	+	+	+	+	+	+	+	+	+^b,c^	-	+	*L. fallax* LMG 18975^a,b^, *L. pseudomesenteroides* LMG 11482 ^T^, *L. kimchii* C2 (partially)
Fused glutamate racemase/NTP pyrophosphatase	+	+	+	+	+	+	+	+	+^b,c^	+	+	Encoded by two separate genes
Source of isolation	Broiler meat [2]	Vegetable sausages [4]	Vegetable salad [9]	Vegetable salad [13]	Fish/carrots [10]	Carrot [10]	Carrot [13]	Carrot [10]	Kimchi [65]	Kimchi [7]	Kimchi [6]	

Strains LMG 18811^T^, KG16-1, C120c, C122c, KSL4-2, PB1a, PB1e, PL111 and LMG 22919 belong to *L. gelidum* subsp. *gasicomitatum*; LMG 18297 ^T^ and JB7 – to *L. gelidum* subsp. *gelidum.* Strains with complete genomes are LMG 18811^T^, KG16-1 and JB7; others have draft genomes. Gene names and locus_tags (either from KG16-1 or LMG 18811 ^T^): lacticin biosynthesis genes *lctA* (LEKG_0458) and *lctMT* (LEKG_0461-0462); polyketide biosynthesis cluster (LEGAS_1827-1830); pyruvate oxidase *poxB* (LEGAS_1053); biofilm formation genes *icaB* and *icaA* (LEGAS_1065, LEGAS_1067); collagen-binding protein *cna* (LEGAS_1063); xylose-proton symporter *xylT* (LEGAS_1062); mucus-binding protein (LEGAS_0414); accessory Sec system genes *secY2, asp1, asp2, asp3, secA2, nss, gtfA, gtfB, asp4* (LEKG_0540-0548); fused glutamate racemase/NTP pyrophosphatase (LEKG_0672)

^a^Phylogenetic analysis (Figure S1 of Additional file 1) indicates that the genome, assigned to *L. inhae* LMG 22919, actually belongs to *L. gelidum* subsp. *gasicomitatum* species

^b^Gene is missing from the genome annotation

^c^Contains frameshift(s)

Overall, the genome set for comparative analysis contained eight *L. gelidum* subsp. *gasicomitatum* genomes (two complete and six draft). Ortholog prediction and subsequent analysis, including pangenome matrix (Additional file 2) construction and identification of group-specific genes, were performed using GET_HOMOLOGUES software package [38] with OrthoMCL clustering algorithm [39] and default parameters (minimum coverage in BLAST pairwise alignments 75 %, maximum e-value 0.00001), except for the minimum sequence identity, which was set to 30 %.

**Fig. 4:**
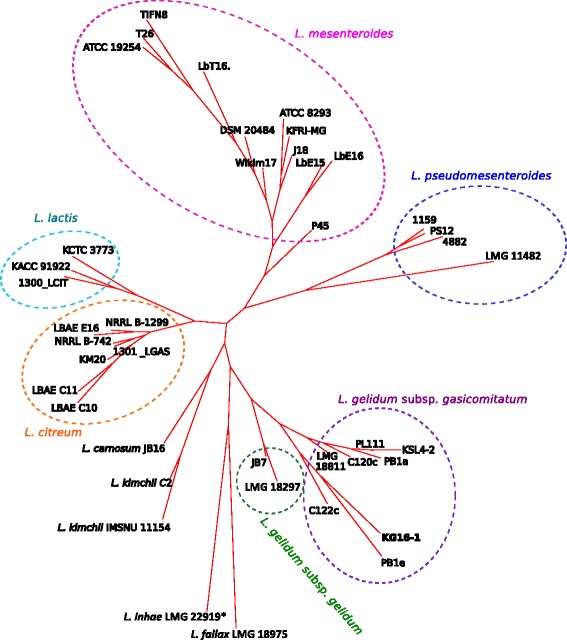
Pangenome tree of the *Leuconostoc* genus, constructed based on information on presence/absence of orthologs. The tree was inferred using PARS program from the PHYLIP package [66] and visualized by the Tree viewer from T-REX web server [61]. *Phylogenetic analysis indicates that the genome assigned to *L. inhae* actually belongs to *L. gelidum* subsp. *gasicomitatum*. The clustering of this genome together with the *L. fallax* LMG 18975T genome was most likely caused by the numerous sequencing errors in this genome, as well as high genome fragmentation

**Fig. 5:**
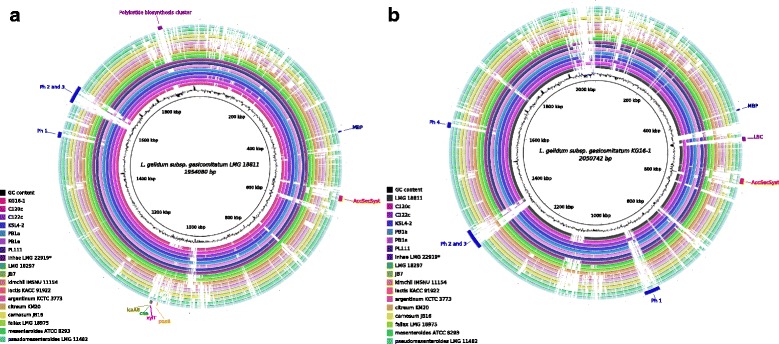
Genome comparisons between *L. gelidum* strains and other leuconostocs. The comparison was done based on the BLASTN alignment and visualized using BRIG [67]. The genomes of *L. gelidum* subsp. *gasicomitatum* LMG 18811^T^ (**a**) and KG16-1 (**b**) were used as references and are represented by the innermost black circles. *L. gelidum* subsp. *gasicomitatum* strains include LMG 18811^T^, KG16-1, C120c, C122c, KSL4-2, PB1a, PB1e, PL111, LMG 22919 (*genome erroneously assigned to *L. inhae* LMG 22919 ^T^) and *L. gelidum* subsp. *gelidum* strains include LMG 18297^T^ and JB7. One genome per *Leuconostoc* species (other than *L. gelidum*) was chosen for the comparisons. The outmost tracks show the location of prophage regions (designated as Ph) and other functionally interesting genes (designations used: MBP - mucus-binding protein LEGAS_0414; LBC - lacticin biosynthesis cluster; AccSecSyst – accessory Sec system; *icaAB -* biofilm formation genes; *cna -* collagen-binding protein; *xylT -* xylose-proton symporter; *poxB* - pyruvate oxidase)

As a result 6,248 orthologous groups were predicted (including singletons) with 406 clusters present in all genomes (so-called core genome), and 983 present in at least 95 % (38) of the genomes (Additional file 2). The last number represents the soft core of the *Leuconostoc* genus, which allowed to account for the missing annotations in draft genomes [40]. The soft core included 52 clusters with unknown function. Based on the pangenome matrix of the presence/absence of the genes in the genomes, a pangenome tree was constructed (Fig. 4). Generally, genomes belonging to the same species clustered together, except for the genomes of *L. gelidum* subsp. *gasicomitatum* 1301_LGAS and *L. citreum* 1300_LCIT obtained during the same study of clinical isolates from the hospital intensive care unit [41], where species names were assigned to the sequenced genomes based on the best matching genome from NCBI database. The first genome clustered together with *L. citreum* strains, while the second genome fell into the same branch with *L. lactis* strains. The phylogenetic analysis (Figure S1 of Additional file 1) placed these genomes into the same branches as in the pangenome tree, thereby confirming that the first genome, formerly assigned to *L. gelidum* subsp. *gasicomitatum* (1301_LGAS), actually belongs to *L. citreum*, while the second genome, assigned to *L. citreum* (1300_LCIT), is a member of the *L. lactis* group. In addition, the phylogenetic analysis (Figure S1 of Additional file 1) showed that the genome assigned to *L. inhae*LMG 22919 (= KCTC 3774) appears to be of *L. gelidum* subsp. *gasicomitatum* strain. It is notable that none of the previously published gene sequences for *L. inhae*LMG 22919 (= KCTC 3774) (16S rRNA [AF439560]; *recN* [GenBank:AM698028]; *recA* [GenBank:JF261010]; *atpA* [GenBank:AM711190]; *pheS* [GenBank:AM711167]; *rpoA* [GenBank:AM711310]) mapped 100 % to the genome assigned to the same *L. inhae* strain [GenBank:GCF_000166735.2]. Instead, the nucleotide sequences of these genes extracted from this genome were identical to those of *L. gelidum* subsp. *gasicomitatum* 18811^T^ [GenBank: GCA_000196855.1]. However, the genome assigned to *L. inhae*LMG 22919 lacked five genomic regions (including two prophages) that are present in *L. gelidum* subsp. *gasicomitatum* 18811^T^ genome, but absent in all or some other *L. gelidum* subsp. *gasicomitatum* strains (Fig. 5a). This might indicate that the considered genome [GenBank:GCF_000166735.2] belongs to the *L. gelidum* subsp. *gasicomitatum* strain, which is very close to strain 18811^T^, but not identical. The clustering of the genome assigned to *L. inhae*LMG 22919 together with *L. fallax* on the pangenome tree (Fig. 4) was, assumingly, the result of numerous frameshifts that are present in this genome (most probably due to the sequencing errors), as well as the high genome fragmentation (893 contigs), which both caused many genes to be missing or truncated in the genome annotation. Although phylogenetically *L. carnosum* was closer to *L. citreum* and *L. lactis* (Fig. 1), in terms of gene content it seemed to be more similar to *L. kimchii* (Fig. 4). Interestingly, there was no clear separation between the meat strain and vegetable strains of *L. gelidum* subsp. *gasicomitatum* based on gene content. On the contrary, five vegetable strains clustered together with the meat strain, while the three remaining vegetable strains formed a separate branch.

*L. gelidum* subsp. *gasicomitatum* KG16-1 genome contained 75 unique genes that are not present in other *Leuconostoc* genomes. Of these genes, uncharacterized and phage protein-coding genes constituted 77 % (58) (Fig. 5b). The genome contained the cluster for lacticin-481 biosynthesis (LEKG_0458-0465), which was also present only in *L. gelidum* subsp. *gasicomitatum* strain C120c and homologies to that in *Lactococcus lactis* subsp. *lactis* [42, 43]. The lacticin-481 type bacteriocin has been shown to be active against LAB and food-spoilage bacterium *Clostridium tyrobutyricum* [43]. Genes involved in catabolism of type I galactan were present in other vegetable strains of *L. gelidum* subsp. *gasicomitatum* besides KG16-1 (KSL4-2, PL111 and PB1e)*.* Peculiarly, the KG16-1 genome was lacking several functionally interesting genes that are present in other *L. gelidum* strains (Table 6, Fig. 5a). They included a polyketide biosynthesis cluster and the genome locus containing pyruvate oxidase *poxB*, collagen-binding protein *cna*, biofilm formation genes *icaAB* and, as already mentioned, xylose transporter *xylT.* Besides seven *L. gelidum* subsp. *gasicomitatum* strains, the same polyketide biosynthesis cluster (in terms of domain architecture and gene synteny) was found only in *Streptococcus thermophilus* JIM 8232 (genes pig-1,2,3,4). Polyketides are bioactive compounds that can exhibit antibacterial, immunosuppressive and antitumor activities [44]. Pyruvate oxidase is a hydrogen peroxide-producing enzyme and was speculated to be associated with meat discoloration [14]. Genes *can* and *icaAB* were suggested to mediate adhesion and, hence, better survival in meat environment of *L. gelidum* subsp. *gasicomitatum*LMG 18811^T^ [14]*.* They exhibit homology to collagen adhesin and polysaccharide adhesin biosynthesis genes, respectively, from *Staphylococcus aureus* [45, 46]*.* Our analysis shows that, except for KG16-1, they were also present in vegetable strains. The orthologs of another putative adhesin, mucus-binding protein (LPxTG-like motif-containing), were found only in *L. gelidum* subsp. *gasicomitatum* strains. In the KG16-1 strain, this gene contained a frameshift due to the insertion of A nucleotide at the position 416,796 (confirmed by PCR and Sanger sequencing). Finally, an accessory Sec system involved in the export and glycosylation of serine-rich adhesins [47] was only detected in a few *Leuconostoc* species, including *L. gelidum**strains* (except for LMG 18297^T^). Serine-rich proteins were found in the vicinity of this system in two complete genomes of *L. gelidum* subsp. *gasicomitatum*LMG 18811^T^ and KG16-1. They were also present in other draft *L. gelidum* subsp. *gasicomitatum* genomes and might be involved in adhesion. The latter might play an important role in the survival and persistence of the bacteria considered in a food-processing environment.

## Conclusions

Seven vegetable spoilage-associated strains of *L. gelidum* subsp. *gasicomitatum* were sequenced, and one (KG16-1) was fully assembled, functionally annotated and described in detail in this paper. The gene contents were compared between these vegetable strains, the meat-spoilage-associated strain LMG 18811^T^ of the same species and 33 other *Leuconostoc* species sequenced to date. As a result, no obvious differences in gene contents between the meat strain and vegetable strains of *L. gelidum* subsp. *gasicomitatum* were found that would explain their adaptation to different ecological niches. Therefore, the absence of cross-contamination between vegetable- and meat-processing chains seems to be the more likely factor explaining strain segregation between vegetable- and meat-based food products. Finally, the distribution of functionally interesting genes (spoilage-, adhesion- and bacteriocin-related) was determined across the *L. gelidum* strains and other leuconostocs.

## Abbreviations

LAB, Lactic acid bacteria; MAP, Modified-atmosphere packaged; MRS, de Man-Rogosa-Sharpe (medium); RM, restriction-modification

## Additional files

Additional file 1: Table S1. Genebank identifiers of the nucleotide sequences of *atpA*, *pheS* and *rpoA* genes, used for the phylogenetic analyses. **Table S2.** NCBI Refseq/Genbank assembly accession numbers for the comparative genomic analysis of *Leuconostoc* species (29.09.2015). **Table S3.** *L. gelidum* subsp. *gasicomitatum* draft genomes statistics. **Figure S1.** Phylogenetic tree showing the relationship of *L. citreum* 1300_LCIT, *L. gelidum* subsp. *gasicomitatum* 1301_LGAS and *L. inhae* LMG 22919 genomes to other *Leuconostoc* species. (PDF 406 kb) Additional file 2: Pangenome matrix for *Leuconostoc* genus, core and softcore genes. (XLSX 1692 kb)

## References

[1] Shaw BG, Harding CD. *Leuconostoc gelidum* sp. nov. and *Leuconostoc carnosum* sp. nov. from chill-stored meats. Int J Syst Bacteriol. 1989;39:217–23.

[2] Björkroth KJ, Geisen R, Schillinger U (2000). Characterization of *Leuconostoc gasicomitatum* sp. nov., associated with spoiled raw tomato-marinated broiler meat strips packaged under modified-atmosphere conditions. Appl Environ Microbiol.

[3] Vihavainen EJ, Björkroth KJ (2007). Spoilage of value-added, high-oxygen modified-atmosphere packaged raw beef steaks by *Leuconostoc gasicomitatum* and *Leuconostoc gelidum*. Int J Food Microbiol.

[4] Vihavainen EJ, Murros AE, Björkroth KJ (2008). *Leuconostoc* spoilage of vacuum-packaged vegetable sausages. J Food Prot.

[5] Pothakos V, Snauwaert C, De Vos P, Huys G, Devlieghere F (2014). Psychrotrophic members of *Leuconostoc gasicomitatum*, *Leuconostoc gelidum* and *Lactococcus piscium* dominate at the end of shelf-life in packaged and chilled-stored food products in Belgium. Food Microbiol.

[6] Jung JY, Lee SH, Jeon CO (2012). Complete genome sequence of *Leuconostoc gelidum* strain JB7, isolated from kimchi. J Bacteriol.

[7] Kim D-S, Choi S-H, Kim D-W (2011). Genome sequence of *Leuconostoc gelidum* KCTC 3527, isolated from kimchi. J Bacteriol.

[8] Rahkila R, De Bruyne K, Johansson P, Vandamme P, Björkroth J (2014). Reclassification of *Leuconostoc gasicomitatum* as *Leuconostoc gelidum* subsp. *gasicomitatum* comb. nov., description of *Leuconostoc gelidum* subsp. *aenigmaticum* subsp. nov., designation of *Leuconostoc gelidum* subsp. *gelidum* subsp. nov. and emended description of *Leuconostoc gelidum*. Int J Syst Evol Microbiol.

[9] Vihavainen EJ, Björkroth KJ (2009). Diversity of *Leuconostoc gasicomitatum* associated with meat spoilage. Int J Food Microbiol.

[10] Lyhs U, Koort JMK, Lundström HS, Björkroth KJ (2004). *Leuconostoc gelidum* and *Leuconostoc gasicomitatum* strains dominated the lactic acid bacterium population associated with strong slime formation in an acetic-acid herring preserve. Int J Food Microbiol.

[11] Pothakos V, Taminiau B, Huys G, Nezer C, Daube G, Devlieghere F (2014). Psychrotrophic lactic acid bacteria associated with production batch recalls and sporadic cases of early spoilage in Belgium between 2010 and 2014. Int J Food Microbiol.

[12] Hultman J, Rahkila R, Ali J, Rousu J, Björkroth KJ (2015). Meat processing plant microbiome and contamination patterns of cold-tolerant bacteria causing food safety and spoilage risks in the manufacture of vacuum-packaged cooked sausages. Appl Environ Microbiol.

[13] Rahkila R, Johansson P, Säde E, Paulin L, Auvinen P, Björkroth J (2015). Multilocus sequence typing of *Leuconostoc gelidum* subsp. *gasicomitatum*, a psychrotrophic lactic acid bacterium causing spoilage of packaged perishable foods. Appl Environ Microbiol.

[14] Johansson P, Paulin L, Säde E, et al. Genome sequence of a food spoilage lactic acid bacterium, *Leuconostoc gasicomitatum* LMG 18811^T^, in association with specific spoilage reactions. Appl Environ Microbiol. 2011;77:4344–51. 10.1128/AEM.00102-11 PMC3127722 21571876

[15] De Bruyne K, Schillinger U, Caroline L (2007). *Leuconostoc holzapfelii* sp. nov., isolated from Ethiopian coffee fermentation and assessment of sequence analysis of housekeeping genes from delineation of *Leuconostoc* species. Int J Syst Evol Microbiol.

[16] Björkroth J, Korkeala H (1996). rRNA gene restriction patterns as a characterization tool for *Lactobacillus sake* strains producing ropy slime. Int J Food Microbiol.

[17] Pitcher DG, Saunders NA, Owen RJ (1989). Rapid extraction of bacterial genomic DNA with guanidium thiocyanate. Lett Appl Microbiol.

[18] Staden R, Beal KF, Bonfield JK (2000). The Staden package, 1998. Methods Mol Biol.

[19] Delcher AL, Bratke KA, Powers EC, Salzberg SL (2007). Identifying bacterial genes and endosymbiont DNA with Glimmer. Bioinformatics.

[20] Hyatt D, Chen G-L, Locascio PF, Land ML, Larimer FW, Hauser LJ (2010). Prodigal: prokaryotic gene recognition and translation initiation site identification. BMC Bioinformatics.

[21] Aziz RK, Bartels D, Best AA (2008). The RAST Server: rapid annotations using subsystems technology. BMC Genomics.

[22] Koskinen JP, Törönen P, Nokso-Koivisto J, Holm L. PANNZER - High-throughput functional annotation of uncharacterized proteins in an error-prone environment. *Bioinformatics.* 2015:doi:10.1093/bioinformatics/btu851. 10.1093/bioinformatics/btu851 25653249

[23] Pati A, Ivanova NN, Mikhailova N (2010). GenePRIMP: a gene prediction improvement pipeline for prokaryotic genomes. Nat Methods.

[24] Antonov I, Borodovsky M (2010). GeneTack: frameshift identification in protein-coding sequences by the Viterbi algorithm. J Bioinform Comput Biol.

[25] Slater GSC, Birney E (2005). Automated generation of heuristics for biological sequence comparison. BMC Bioinformatics.

[26] de Jong A, van Heel AJ, Kok J, Kuipers OP (2010). BAGEL2: mining for bacteriocins in genomic data. Nucleic Acids Res.

[27] Zhou Y, Liang Y, Lynch KH, Dennis JJ, Wishart DS (2011). PHAST: a fast phage search tool. Nucleic Acids Res.

[28] Grissa I, Vergnaud G, Pourcel C (2007). CRISPRFinder: a web tool to identify clustered regularly interspaced short palindromic repeats. Nucleic Acids Res.

[29] Lagesen K, Hallin P, Rødland EA, Staerfeldt H-H, Rognes T, Ussery DW (2007). RNAmmer: consistent and rapid annotation of ribosomal RNA genes. Nucleic Acids Res.

[30] Lowe TM, Eddy SR (1997). tRNAscan-SE: A program for improved detection of transfer RNA genes in genomic sequence. Nucleic Acids Res.

[31] Laslett D, Canback B (2004). ARAGORN, a program to detect tRNA genes and tmRNA genes in nucleotide sequences. Nucleic Acids Res.

[32] Krogh A, Larsson B, von Heijne G, Sonnhammer EL (2001). Predicting transmembrane protein topology with a hidden Markov model: application to complete genomes. J Mol Biol.

[33] Petersen TN, Brunak S, von Heijne G, Nielsen H (2011). SignalP 4.0: discriminating signal peptides from transmembrane regions. *Nat*. Methods.

[34] Klimke W, O’Donovan C, White O (2011). Solving the problem: genome annotation standards before the data deluge. Stand Genomic Sci.

[35] Park I, Walsh CT. D-alanyl-D-lactate and D-alanyl-D-alanine synthesis by D-alanyl-D-alanine ligase from vancomycin-resistant *Leuconostoc mesenteroides*. J Biol Chem. 1997;272:9210–4. 10.1074/jbc.272.14.9210 9083053

[36] Shipkowski S, Brenchley JE (2006). Bioinformatic, genetic, and biochemical evidence that some glycoside hydrolase family 42 beta-galactosidases are arabinogalactan type I oligomer hydrolases. Appl Environ Microbiol.

[37] Zerbino DR, Birney E (2008). Velvet: algorithms for de novo short read assembly using de Bruijn graphs. Genome Res.

[38] Contreras-Moreira B, Vinuesa P. GET_HOMOLOGUES, a versatile software package for scalable and robust microbial pan-genome analysis. Appl Environ Microbiol. 2013;79. 10.1128/AEM.02411-13 PMC3837814 24096415

[39] Li L, Stoeckert CJ, Roos DS (2003). OrthoMCL: identification of ortholog groups for eukaryotic genomes. Genome Res.

[40] Kaas RS, Friis C, Ussery DW, Aarestrup FM (2012). Estimating variation within the genes and inferring the phylogeny of 186 sequenced diverse *Escherichia coli* genomes. BMC Genomics.

[41] Roach DJ, Burton JN, Lee C (2015). A year of infection in the intensive care unit: prospective whole genome sequencing of bacterial clinical isolates reveals cryptic transmissions and novel microbiota. PLoS Genet.

[42] Rince A, Dufour A, Uguen P, Pennec JLE, De Ge L. Characterization of the lacticin 481 operon: the *Lactococcus lactis* genes *lctF, lctE*, and *lctG* encode a putative ABC transporter involved in bacteriocin immunity. Appl Environ Microbiol. 1997;63:4252–60. 10.1128/aem.63.11.4252-4260.1997 PMC168744 9361411

[43] Piard JC, Muriana PM, Desmazeaud MJ, Klaenhammer TR (1992). Purification and partial characterization of lacticin 481, a lanthionine-containing bacteriocin produced by *Lactococcus lactis* subsp. *lactis* CNRZ 481. Appl Environ Microbiol.

[44] Gokhale RS, Sankaranarayanan R, Mohanty D (2007). Versatility of polyketide synthases in generating metabolic diversity. Curr Opin Struct Biol.

[45] Patti JM, Jonsson H, Guss B (1992). Molecular characterization and expression of a gene encoding a *Staphylococcus aureus* collagen adhesin. J Biol Chem.

[46] Cramton SE, Gerke C, Schnell NF, Nichols WW, Götz F (1999). The intercellular adhesion (*ica*) locus is present in *Staphylococcus aureus* and is required for biofilm formation. Infect Immun.

[47] Feltcher ME, Braunstein M (2012). Emerging themes in SecA2-mediated protein export. Nat Rev Microbiol.

[48] Field D, Garrity G, Gray T (2008). The minimum information about a genome sequence (MIGS) specification. Nat Biotechnol.

[49] Woese CR, Kandler O, Wheelis ML (1990). Towards a natural system of organisms: proposal for the domains Archaea, Bacteria, and Eucarya. Proc Natl Acad Sci U S A.

[50] Gibbons NE, Murray RGE (1978). Proposals concerning the higher taxa of Bacteria. Int J Syst Bacteriol.

[51] Garrity G, Holt J, Boone D, Castenholz R, Garrity G (2001). The Road Map to the Manual. Bergey’s Manual of Systematic Bacteriology.

[52] Ludwig W, Schleifer K-H, Whitman WB, De Vos P, Garrity G, Jones D (2009). Class I. *Bacilli* class nov. Bergey’s Manual of Systematic Bacteriology.

[53] Ludwig W, Schleifer K-H, Whitman WB, De Vos P, Garrity G, Jones D, De Vos P, Garrity G, Jones D (2009). Order II. *Lactobacillales* ord. nov. Bergey’s Manual of Systematic Bacteriology.

[54] Schleifer K-H, Family V, De Vos P, Garrity G, Jones D (2009). *Leuconostocaceae* fam. nov. Bergey’s Manual of Systematic Bacteriology.

[55] van Tieghem P (1878). Sur la gomme de sucrerie (*Leuconostoc mesenteroides*). Ann des Sci Nat Bot.

[56] Garvie EI, Sneath, Mair, Sharpe, Holt (1986). Genus *Leuconostoc* van Tieghem 1878, 198AL emend. mut. char. Hucker and Pederson 1930, 66AL. Bergey’s Manual of Systematic Bacteriology.

[57] Skerman V, McGowan V, Sneath P (1980). Approved lists of bacterial names. Int J Syst Bacteriol.

[58] Nieminen T, Sade E, Endo A, Johansson P, Bjorkroth J, Rosenberg E, DeLong E, Lory S, Stackebrandt E, Thompson F (2014). The Family Leuconostocaceae. The Prokaryotes – Firmicutes and Tenericutes.

[59] Björkroth J, Holzapfel W, Dworkin M (2006). Genera *Leuconostoc, Oenococcus* and *Weissella*. The prokaryotes: a handbook on the biology of bacteria: Firmicutes, Cyanobacteria.

[60] Ashburner M, Ball CA, Blake JA (2000). Gene Ontology : tool for the unification of biology. Nat Genet.

[61] Boc A, Diallo AB, Makarenkov V (2012). T-REX: a web server for inferring, validating and visualizing phylogenetic trees and networks. Nucleic Acids Res.

[62] Edgar RC (2004). MUSCLE: multiple sequence alignment with high accuracy and high throughput. Nucleic Acid Res.

[63] Castresana J (2000). Selection of conserved blocks from multiple alignments for their use in phylogenetic analysis. Mol Biol Evol.

[64] Stamatakis A (2006). RAxML-VI-HPC: maximum likelihood-based phylogenetic analyses with thousands of taxa and mixed models. Bioinformatics.

[65] Kim D-S, Choi S-H, Kim D-W (2011). Genome sequence of *Leuconostoc inhae* KCTC 3774, isolated from kimchi. J Bacteriol.

[66] Felsenstein J (1989). PHYLIP - Phylogeny Inference Package (Version 3.2). Cladistics.

[67] Alikhan N-F, Petty NK, Ben Zakour NL, Beatson SA (2011). BLAST Ring Image Generator (BRIG): simple prokaryote genome comparisons. BMC Genomics.

