# Supplementary material for: Complete genome sequence of Leuconostoc gelidum subsp. gasicomitatum KG16-1, isolated from vacuum-packaged vegetable sausages
Source: Stand Genomic Sci. 2016 Jun 7;11:40. doi: 10.1186/s40793-016-0164-8 (PMC4895993; doi:10.1186/s40793-016-0164-8)
Supplement: Additional file 1: Table S1. — Genebank identifiers of the nucleotide sequences of atpA, pheS and rpoA genes, used for the phylogenetic analyses. Table S2. NCBI Refseq/Genbank assembly accession numbers for the comparative genomic analysis of Leuconostoc species (29.09.2015). Table S3. L. gelidum subsp. gasicomitatum draft genomes statistics. Figure S1. Phylogenetic tree showing the relationship of L. citreum 1300_LCIT, L. gelidum subsp. gasicomitatum 1301_LGAS and L. inhae LMG 22919 genomes to other Leuconostoc species. (PDF 406 kb) [file 40793_2016_164_MOESM1_ESM.pdf]

Additional file 1

Table S1. Genebank identifiers of the nucleotide sequences of *atpA*, *pheS* and *rpoA* genes, used for the phylogenetic analyses.

Species	*atpA*	*pheS*	*rpoA*
Bacillus subtilis 168^T	NC_000964	NC_000964	NC_000964
L. carnosum LMG 23898^T	AM711275	AM711282	AM711353
L. citreum LMG 9849^T	AM711202	AM711152	AM711300
L. fallax LMG 18975^T	AM711284	AM711193	AM711343
L. gelidum subsp. *aenigmaticum* POKY4-4	KF577554	KF577556	KF577561
L. gelidum subsp. *aenigmaticum* POUF4d^T	KF577549	KF577560	KF577564
L. gelidum subsp. *gasicomitatum* LMG 18811^T	AM711180	AM711150	AM711318
L. gelidum subsp. *gasicomitatum* LMG 18813	AM711201	AM711162	AM711317
L. gelidum subsp. *gasicomitatum* KG16-1	LN890331	LN890331	LN890331
L. gelidum subsp. *gelidum* LMG 18297^T	AM711204	AM711160	AM711307
L. gelidum subsp. *gelidum* JB7	CP003839	CP003839	CP003839
L. holzapfelii LMG 23990^T	AM711273	AM711209	AM711354
L. inhae LMG 22919^T	AM711190	AM711167	AM711310
L. kimchii LMG 23786^T	AM711220	AM711195	AM711339
L. lactis LMG 8894^T	AM711253	AM711267	AM711311
L. mesenteroides subsp. *mesenteroides* LMG 6893^T	AM711176	AM711145	AM711294
L. pseudomesenteroides LMG 11482^T	AM711175	AM711197	AM711315
L. citreum 1300_LCIT*	NZ_JVUV01000032	NZ_JVUV01000047	NZ_JVUV01000055
L. gelidum subsp. *gasicomitatum* 1301_LGAS**	NZ_JVUU01000022	NZ_JVUU01000031	NZ_JVUU01000023
L. inhae LMG 22919 (= KCTC 3774, genome)***	NZ_AEMJ01000006	NZ_AEMJ01000675	NZ_AEMJ01000861
L. citreum KM20	NC_010471	NC_010471	NC_010471
L. argentinum (later synonym of *L. lactis* [1]) KCTC 3773	NZ_AEGQ01000052	NZ_AEGQ01000042	NZ_AEGQ01000003
L. lactis KACC 91922	NZ_JMEA01000009	NZ_JMEA01000007	NZ_JMEA01000004

* Phylogenetic and pangenome analyses indicate the belonging of this genome to *L. lactis* species (see the main body of the article).
** Phylogenetic and pangenome analyses indicate the belonging of this genome to *L. citreum* species (see the main body of the article).
*** Phylogenetic analysis indicates the belonging of this genome to *L. gelidum* subsp. *gasicomitatum* species (see the main body of the article).

Table S2. NCBI assembly accession numbers/ INSDC identifiers for the comparative genomic analysis of *Leuconostoc* species (29.09.2015).

Species	NCBI assembly accession number/ INSDC identifiers
Leuconostoc argentinum (later synonym of *L. lactis* [1]) KCTC 3773	GCF_000179875.1
Leuconostoc carnosum JB16	GCF_000300135.1
Leuconostoc citreum NRRL B-742	GCF_000820965.1
Leuconostoc citreum NRRL B-1299	GCF_000820985.1
Leuconostoc citreum KM20	GCF_000026405.1
Leuconostoc citreum LBAE C10	GCF_000239895.2
Leuconostoc citreum LBAE C11	GCF_000239915.2
Leuconostoc citreum LBAE E16	GCF_000239935.2
Leuconostoc citreum 1300_LCIT*	GCF_001062635.1
Leuconostoc fallax LMG 18975^{T} (= KCTC 3537)	GCF_000165675.2
Leuconostoc gelidum subsp. *gasicomitatum* LMG 18811 ^{T}	GCA_000196855.1
Leuconostoc gelidum subsp. *gasicomitatum* KG16-1	LN890331- LN890334
Leuconostoc gelidum subsp. *gasicomitatum* C120c	FBTC01000001-FBTC01000031
Leuconostoc gelidum subsp. *gasicomitatum* C122c	FBSY01000001-FBSY01000022
Leuconostoc gelidum subsp. *gasicomitatum* KSL4-2	FBTB01000001-FBTB01000023
Leuconostoc gelidum subsp. *gasicomitatum* PB1a	FBSX01000001-FBSX01000029
Leuconostoc gelidum subsp. *gasicomitatum* PB1e	FBTD01000001-FBTD01000022
Leuconostoc gelidum subsp. *gasicomitatum* PL111	FBTU01000001-FBTU01000029
Leuconostoc gelidum subsp. *gasicomitatum* 1301_LGAS**	GCF_001077275.1
Leuconostoc gelidum subsp. *gelidum* JB7	GCF_000298875.1
Leuconostoc gelidum subsp. *gelidum* LMG 18297 ^{T} (= KCTC 3527)	GCF_000166715.2
Leuconostoc inhae LMG 22919 (= KCTC 3774)***	GCF_000166735.2
Leuconostoc kimchii IMSNU 11154	GCF_000092505.1
Leuconostoc kimchii C2	GCF_000219785.1
Leuconostoc lactis KACC 91922	GCF_000709265.1
Leuconostoc mesenteroides Wikim17	GCF_000787735.1
Leuconostoc mesenteroides KFRI-MG	GCF_000512955.1
Leuconostoc mesenteroides P45	GCF_000756355.1
Leuconostoc mesenteroides subsp. *cremoris* LbT16	GCF_001184265.1
Leuconostoc mesenteroides subsp. *cremoris* ATCC 19254	GCF_000160595.1
Leuconostoc mesenteroides subsp. *cremoris* T26	GCF_000686485.1
Leuconostoc mesenteroides subsp. *cremoris* TIFN8	GCF_000447945.1
Leuconostoc mesenteroides subsp. *dextranicum* DSM 20484	GCF_001047695.1
Leuconostoc mesenteroides subsp. *dextranicum* LbE15	GCF_001184245.1
Leuconostoc mesenteroides subsp. *mesenteroides* LbE16	GCF_001184255.1
Leuconostoc mesenteroides subsp. *mesenteroides* ATCC 8293	GCF_000014445.1
Leuconostoc mesenteroides subsp. *mesenteroides* J18	GCF_000234825.2
Leuconostoc pseudomesenteroides 1159	GCF_000686465.1
Leuconostoc pseudomesenteroides 4882	GCF_000297375.1
Leuconostoc pseudomesenteroides LMG 11482 ^{T} (= KCTC 3652)	GCF_000185065.2
Leuconostoc pseudomesenteroides PS12	GCF_000686505.1

* Phylogenetic and pangenome analyses indicate the belonging of this genome to *L. lactis* species (see the main body of the article).
** Phylogenetic and pangenome analyses indicate the belonging of this genome to *L. citreum* species (see the main body of the article).
*** Phylogenetic analysis indicates the belonging of this genome to *L. gelidum* subsp. *gasicomitatum* species (see the main body of the article).

Table S3. *L. gelidum* subsp. *gasicomitatum* draft genomes statistics.

Strain	Genome size, Mb	Fold coverage	No of contigs	No of CDSs	Locus Tag	INSDC identifiers
**C120c**	2,00	242	31	1962	C120C	FBTC01000001-FBTC01000031
**C122c**	2,01	631	22	2006	C122C	FBSY01000001-FBSY01000022
**KSL4-2**	2,01	1634	23	2085	KSL4	FBTB01000001-FBTB01000023
**PB1a**	2,09	439	29	2061	PB1A	FBSX01000001-FBSX01000029
**PB1e**	2,09	1034	22	2107	PB1E	FBTD01000001-FBTD01000022
**PL111**	2,06	418	29	2020	PL111	FBTU01000001-FBTU01000029

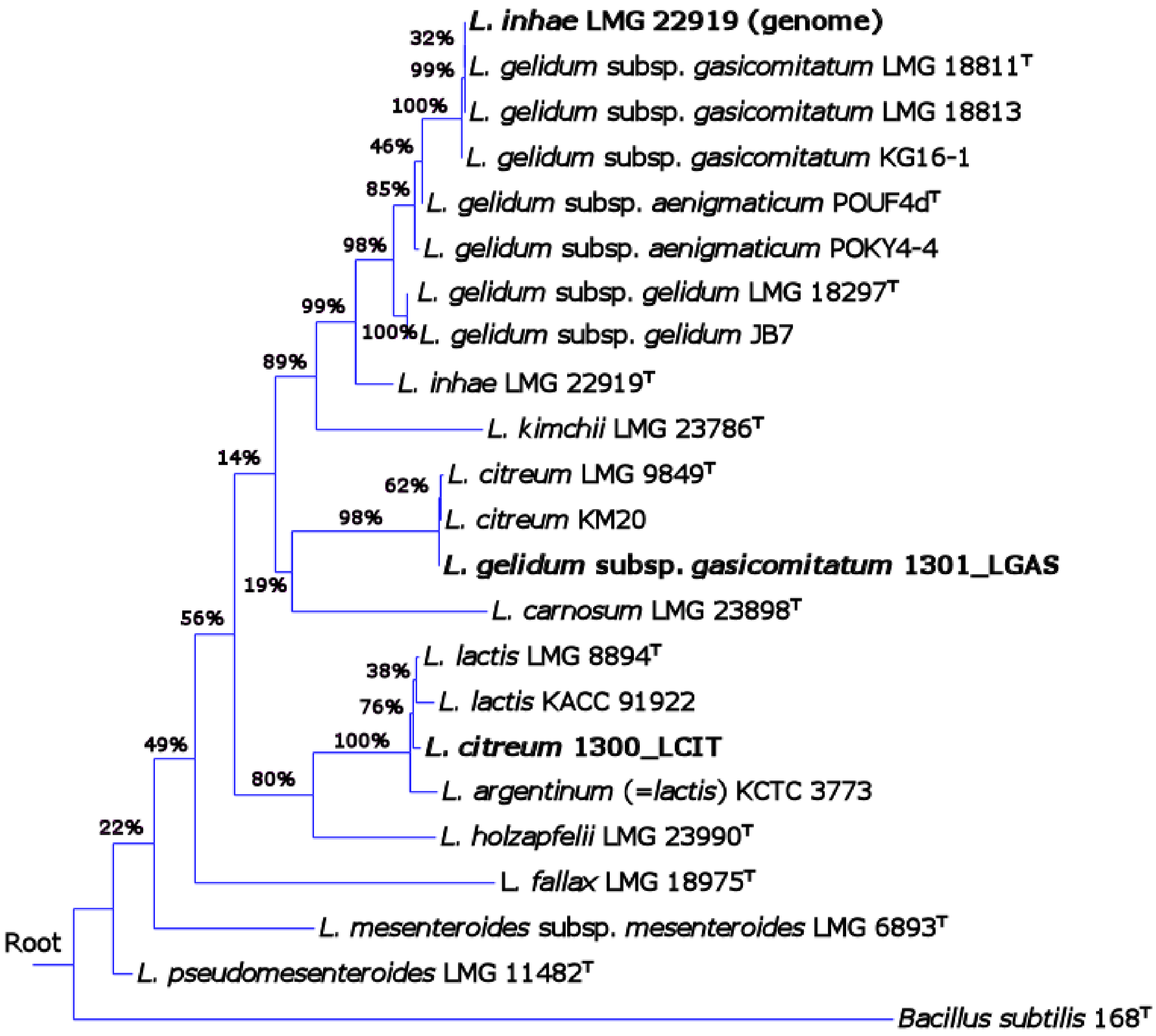

Figure S1. Phylogenetic tree showing the relationship of *L. citreum* 1300_LCIT, *L. gelidum* subsp. *gasicomitatum* 1301_LGAS and *L. inhae* LMG 22919 genomes to other *Leuconostoc* species. The tree was built using T-REX web server [2] based on MUSCLE [3] aligned concatenated nucleotide sequences of *atpA*, *pheS* and *rpoA* genes (Genbank identifiers are listed in the Table S1 of Additional file 1). Poorly aligned positions were removed using Gblocks [4] with default parameters and the tree was inferred using RAxML program [5] with GTRCAT substitution model and *Bacillus subtilis* as an outgroup. Rapid bootstrap analysis was performed with 500 replicates. Type strains are identified with superscripted “T”.

References

1. Vancanneyt M, Zamfir M, De Wachter M, *et al.* Reclassification of *Leuconostoc argentinum* as a later synonym of *Leuconostoc lactis*. *Int. J. Syst. Evol. Microbiol.* 2006;**56**:213–6.

2. Boc A, Diallo AB, Makarenkov V. T-REX: a web server for inferring, validating and visualizing phylogenetic trees and networks. *Nucleic Acids Res.* 2012;**40**:W573–W579.

3. Edgar RC. MUSCLE: multiple sequence alignment with high accuracy and high throughput. *Nucleic Acid Res.* 2004;**32**:1792–1797.

4. Castresana J. Selection of conserved blocks from multiple alignments for their use in phylogenetic analysis. *Mol. Biol. Evol.* 2000;**17**:540–552.

5. Stamatakis A. RAxML-VI-HPC: maximum likelihood-based phylogenetic analyses with thousands of taxa and mixed models. *Bioinformatics* 2006;**22**:2688–2690.
